# A gut feeling for drugs that have metabolic benefits

**DOI:** 10.1038/s41467-023-40167-3

**Published:** 2023-07-25

**Authors:** Eryun Zhang, Alon Agua, Wendong Huang

**Affiliations:** 1 Department of Diabetes Complications and Metabolism, Arthur Riggs Diabetes and Metabolism Research Institute, Beckman Research Institute, City of Hope National Medical Center, 1500 E. Duarte Road, Duarte, CA 91010 USA; 2 Irell & Manella Graduate School of Biomedical Science, City of Hope National Medical Center, 1500 E. Duarte Road, Duarte, CA 91010 USA

**Keywords:** Translational research, Microbiota, Endocrine system and metabolic diseases

## Abstract

Resveratrol (REV) is a natural polyphenol with anti-obesity effects. However, the mechanisms remain unclear due to its low bioavailability and the lack of defined membrane-bound or nuclear receptors. Pang and colleagues reported that REV intervention (REV-I) alters gut microbiota and bile acid profile, leading to the inhibition of farnesoid X receptor (FXR) and attenuation of scavenger receptor class B type 1 (SR-B1)-mediated chylomicron secretion. This highlights a therapeutic potential of targeting gut microbiome and intestinal SR-B1 for obesity and diabetes treatment.

Resveratrol (REV) is a natural polyphenol with anti-obesity and anti-diabetes effects. Studies demonstrated the profound effects of resveratrol on improving insulin signaling and alleviating dyslipidemia. However, the mechanisms underlying these beneficial effects have not been fully elucidated, in part, due to its poor bioavailability. Intriguingly, after oral administration, 80% of REV reached the gut and remained in the intestinal mucosa of mice for 24 h^1^. This suggests that the gut is a primary site of REV action. Moreover, REV, acting in the duodenum via a neuronal network and dSirt1, improved insulin sensitivity and lowering hepatic glucose production in obese diabetic rats^2^.

Interestingly, REV administration led to marked changes in the composition of the gut microbiota in obese mice, which were characterized by a decreased relative abundance of *Turicibacteraceae*, *Moryella*, *Lachnospiraceae*, and *Akkermansia* and an increased relative abundance of *Bacteroides* and *Parabacteroides*^3^. Transplantation of the fecal matter from REV-fed mice was sufficient to recapitulate the improvement in glucose regulation observed in mice that received oral REV^2,3^. Together, the data suggest that alterations of gut microbiota are important in beneficial effects of REV. Thus, studies that continue to explore how REV does such useful things are welcome.

## REV-1 targets gut microbiota/CDCA/SR-B1 axis to inhibit chylomicron secretion

In this journal, Pang and colleagues showed that high fat diet (HFD) challenged mice given REV-I for 8 weeks had less chylomicron secretion, as determined by reduced triglyceride (TG) content and ApoB48 levels in isolated TG-rich lipoprotein (TRL)^4^. Furthermore, REV-I reduced the expression of scavenger receptor class B type 1 (SR-B1) in the jejunum but not the liver. To further link gut SR-B1 and REV-I effects, experiments were performed in intestinal mucosa-specific SR-B1 KO (iScarb1^−/−^) mice. Here too, an 8-week course of REV-I reduced body weight gain, improved glucose, and insulin tolerance in HFD-fed iScarb1^fl/fl^ but not iScarb1^−/−^ mice. Importantly, after eating an HFD, iScarb1^−/−^ mice showed lower fasting and postprandial plasma TG accumulation and lower TG and ApoB48 levels in TRL. However, REV-I generated no further reduction in these parameters in iScarb1^−/−^ mice. These results suggested that gut-localized SR-B1 is a target of REV-I to reduce chylomicron secretion and improve energy homeostasis.

Interestingly, repression of SR-B1 and chylomicron secretion were not observed in Caco-2 cells exposed to the pure resveratrol compound. However, Caco-2 cells incubated with sterile fecal extract from mice on an HFD plus REV-I showed significantly lower levels of SR-B1 and chylomicron secretion than cells incubated with fecal extract from HFD-fed mice not treated with REV. This result suggests that the inhibitory effect of REV-1 on SR-B1 involves the gut microbiota. Fecal microbiota transplantation (FMT) from REV-1 treated mice to HFD mice, as compared to FMT from vehicle-treated mice, lowered postprandial TG levels and reduced jejunal SR-B1 expression. 16 S ribosomal DNA sequencing revealed that REV-I reduced the density of bile salt hydrolase (BSH)-producing microbiomes, including *Lactobacillus*, *Bifidobacterium*, *Clostridium*, and *Enterococcus*. This in turn was associated with reduced fecal BSH activity and reduced fecal chenodeoxycholic acid (CDCA) level. BSH catalyzes the hydrolysis of conjugated bile salts into de-conjugated bile acids, including CDCA. Importantly, CDCA, a natural farnesoid X receptor (FXR) agonist, upregulated SR-B1 transcription in Caco-2 cells. Furthermore, Gug (Guggulsterone, a FXR inhibitor) blocked CDCA-induced expression of SR-B1 in Caco-2 cells, which suggested FXR was sufficient for CDCA to increase gut SR-B1 level. Predictably, extended treatment with GW4064, a potent FXR agonist, further increased postprandial TG, TRL-TG, and TRL-ApoB48 levels, as well as jejunal, but not hepatic, SR-B1 expression in HFD-fed mice. GW4064 also blocked the inhibitory effects of REV-I on those parameters. Taken together, these results pointed to a gut micriobiota-CDCA-FXR-SR-B1 signaling cascade, which contributes to the metabolic effects of REV-I.

## Perspective and future directions

The gut microbiota regulates host metabolism and gut microbial dysbiosis is associated with obesity and diabetes. Bile acids (BAs) are host-derived and microbial-modified metabolites that play important roles in regulating glucose and lipid homeostasis. The primary BAs, cholic acid (CA) and CDCA in people, or α/β-muricholic acid (α/βMCA) in mice, are synthesized from cholesterol in the liver, conjugated with glycine or taurine, and then released into the intestine to facilitate the absorption of nutrients. Gut microbes metabolize BAs into a variety of different derivatives. For example, conjugated BAs is de-conjugated through microbial BSH and primary BAs such as CDCA are transformed by microbial 7α-hydroxysteroid dehydrogenase (7α-HSDH) into secondary BAs such as ursodeoxycholic acid (UDCA), or by 7α-dehydroxylase to form lithocholic acid (LCA). This bacterial metabolism changes the bioavailability and bioactivities of BAs, and consequently their impact on the metabolic responses. Takeda G protein-coupled receptor 5 (TGR5) and FXR are BA receptors that mediate the BA effects on lipid and glucose homeostasis. Both FXR and TGR5 regulate glucagon-like peptide-1 (GLP-1) levels and modulate glucose metabolism. Conversely, BAs can modulate gut microbial composition both directly and indirectly through activation of innate immune genes in the intestine^5^. Thus, host metabolism can be affected through microbial modifications of BAs, which lead to altered signaling via BA receptors, as well as by altered microbiota composition.

Antidiabetic medications such as metformin, curcumin, and berberine exert beneficial effects, in part, by altering gut microbiome symbiosis and microbial BA metabolism. Metagenomic and metabolomic analysis of intestinal contents from individuals with newly diagnosed type 2 diabetes (T2D) treated with metformin found increased levels of glycoursodeoxycholic acid (GUDCA) in the gut and decreased abundance of *B. fragilis*^6^. BSH activity was also down accompanied by inhibition of intestinal FXR signaling which suggested that the metformin improvement of metabolism was mediated by a *B. fragilis*–GUDCA–intestinal FXR axis. They further found *B. fragilis* colonization exacerbated metabolic disorders induced by a HFD regimen. Moreover, improvements of glucose metabolism in metformin-treated mice were reversed by *B. fragilis*. Shin et al.^7^ found that HFD-fed mice treated with metformin showed a higher abundance of the mucin-degrading bacterium Akkermansia than HFD-fed control mice did. Oral administration of Akkermansia muciniphila to HFD-fed mice without metformin significantly enhanced glucose tolerance and attenuated adipose tissue inflammation. The identification and intervention of key intestinal microbes and their metabolic enzymes play a key role in proving the function of intestinal microbes and have important clinical translational values. Curcumin promoted Ucp1-dependent thermogenesis and ameliorated HFD-induced obesity, a process influenced by the gut microbiota^8^. In addition, curcumin altered BA metabolism with increased fractions of circulating deoxycholic acid (DCA) and LCA, both of them are ligands for TGR5^8^. BBR treatment altered the gut microbiota composition, inhibited the DCA biotransformation by *Ruminococcus bromii* and lowered gut FXR activity^9^. Interestingly, BA signaling was shown to mediate the therapeutic effects of caloric restrictions (CR)^10^. CR altered the microbiota, resulting in a distinct serum bile acid profile characterized by less non-12α-hydroxylated bile acids, UDCA and LCA^10^. Thus, the gut microbiome and BA signaling, in particular, may find a place in the treatment of T2D.

Other microbial metabolites such as short-chain fatty acids (SCFAs), branched chain amino acids, succinate, indole propionic acid, and endocannabinoids were implicated in mediating bacterial regulation of host metabolism^11,12^. Zhuang et al. reported that REV-1 supplementation increased abundance of *Butyricicoccus*, *Ruminococcus_1*, and *Roseburia*, which are producers of SCFAs, in mice feces^13^. Likewise, metformin treatment in individuals with T2D significantly altered circulating SCFAs, including increasing acetate, which was correlated with lower fasting insulin^14^. Intestinal AMPKα1 deficiency altered energy expenditure and brown fat thermogenesis via changes in the gut microbiome and metabolites, particularly methylglyoxal^15^. The circulating methylglyoxal levels were significantly decreased in the metformin-treated AMPKα1^fl/fl^, but not AMPKα1-IKO mice.

Despite this, some important questions remain for future research. For example, how is the gut microbiome involved in BA homeostasis in response to REV-I, including BA synthesis in the liver, gut modifications, and enterohepatic circulation? Further investigations are also needed to determine the relationships between CDCA-FXR and SR-B1 in complicated physiological and pathophysiological settings. An intestinal FXR tissue-specific mouse line will assist in delineating the roles of intestinal FXR related to REV-1 effects. Finally, human and rodent BAs have substantially different species and signaling properties, which calls for additional attention for proper interpretation and translational conclusions.

**Fig. 1:**
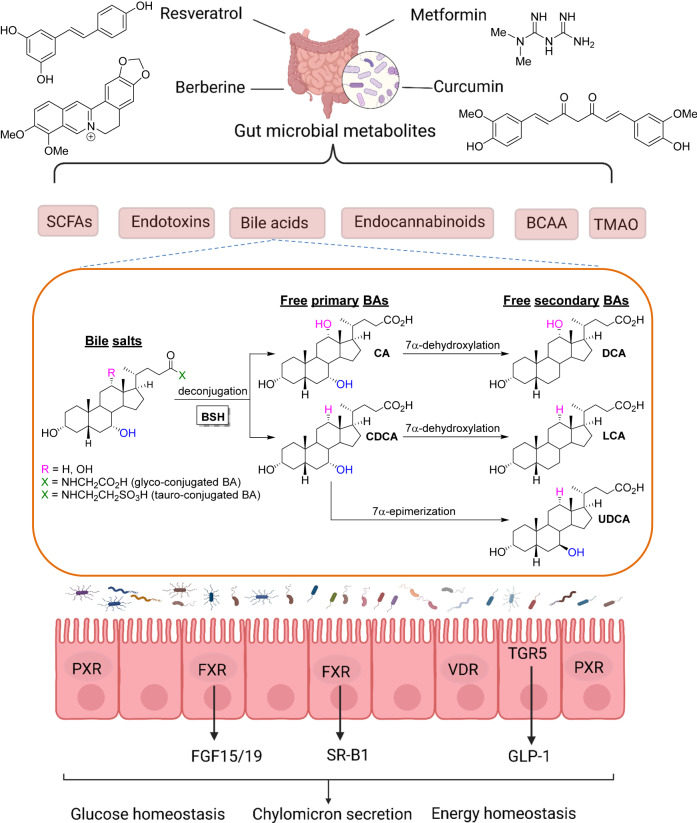
A prospective unified mechanism by which gut microbiome and bile acids regulate metabolism. Resveratrol, metformin, berberine, and curcumin affect the gut microbiota, which results in host metabolic homeostasis via microbial metabolites production, including SCFAs, endotoxins, bile acids (BAs), endocannabinoids, BCAA, and TMAO. The primary BAs, cholic acid (CA) and chenodeoxycholic acid (CDCA) in humans, or α/β-muricholic acid (α/βMCA) in mice, are synthesized from cholesterol in the liver, conjugated with glycine or taurine, and drain into the intestine to facilitate the absorption of nutrients. Within the intestinal lumen, conjugated primary bile acids are metabolized by bile salt hydrolase (BSH) from gut bacteria, which deconjugates and reconverts the conjugated primary BAs into free primary BAs CA and CDCA. The multistep 7a-dehydroxylation pathway continues to perform dehydroxylation reactions on CA and CDCA to form secondary BAs. Namely, CA is converted into deoxycholic acid (DCA) and CDCA is converted into lithocholic acid (LCA) and ursodeoxycholic acid (UDCA). These BAs target BA receptors and related signaling pathways, which results in glucose homeostasis, chylomicron secretion, and energy homeostasis.

In summary, the study by Pang and colleagues^4^ uncovers a new avenue for determining a prospective unified mechanism by which dietary polyphenols and other drugs exert their metabolic effects upon the gut microbiota and BA signaling cascades (Fig. 1).
